# Current Affairs of Microbial Genome-Wide Association Studies: Approaches, Bottlenecks and Analytical Pitfalls

**DOI:** 10.3389/fmicb.2019.03119

**Published:** 2020-01-30

**Authors:** James Emmanuel San, Shakuntala Baichoo, Aquillah Kanzi, Yumna Moosa, Richard Lessells, Vagner Fonseca, John Mogaka, Robert Power, Tulio de Oliveira

**Affiliations:** ^1^Kwazulu-Natal Research and Innovation Sequencing Platform (KRISP), College of Health Sciences, University of KwaZulu-Natal, Durban, South Africa; ^2^Department of Digital Technologies, FoICDT, University of Mauritius, Réduit, Mauritius; ^3^Laboratório de Genética Celular e Molecular, ICB, Universidade Federal de Minas Gerais, Belo Horizonte, Brazil; ^4^Discipline of Public Health, University of Kwazulu-Natal, Durban, South Africa; ^5^St Edmund Hall, Oxford University, Oxford, United Kingdom; ^6^Department of Global Health, University of Washington, Seattle, WA, United States

**Keywords:** microbial genome-wide association studies, microbial GWAS tools and methods, variant analysis, genotype-phenotype association, NGS analysis, SNPs

## Abstract

Microbial genome-wide association studies (mGWAS) are a new and exciting research field that is adapting human GWAS methods to understand how variations in microbial genomes affect host or pathogen phenotypes, such as drug resistance, virulence, host specificity and prognosis. Several computational tools and methods have been developed or adapted from human GWAS to facilitate the discovery of novel mutations and structural variations that are associated with the phenotypes of interest. However, no comprehensive, end-to-end, user-friendly tool is currently available. The development of a broadly applicable pipeline presents a real opportunity among computational biologists. Here, (i) we review the prominent and promising tools, (ii) discuss analytical pitfalls and bottlenecks in mGWAS, (iii) provide insights into the selection of appropriate tools, (iv) highlight the gaps that still need to be filled and how users and developers can work together to overcome these bottlenecks. Use of mGWAS research can inform drug repositioning decisions as well as accelerate the discovery and development of more effective vaccines and antimicrobials for pressing infectious diseases of global health significance, such as HIV, TB, influenza, and malaria.

## Introduction

Microbial genome-wide association studies (mGWAS) are a new area of research aimed at identifying genetic variants in microbial genomes that are associated with host variation in or microbe phenotypes, for example genetic variation affecting phenotypes such as carriage (Lees et al., 2017) in humans and virulence (Laabei, 2014) in microbes. It has also been applied to determine genes responsible for species-specific phenotypes in *Helicobacter pylori* (Dutilh et al., 2013) and to evaluate interactions between host and microbe genomes (Bartha et al., 2013).

Successful applications of mGWAS include identifying genetic determinants of pyomyositis in *Staphylococcus aureus* (Young et al., 2019) which revealed that the presence of Panton-Valentine leucocidin (PVL) locus increased the odds of pyomyositis. In another study, (Lees et al., 2019) showed that variations in *Streptococcus pneumoniae* explain large amounts of the invasiveness potential but have no effect on severity of pneumococcal meningitis. Furthermore, mGWAS was used by Davies et al. (2019) to determine vaccine candidate coverage from 2083 Group A *Streptococcus* (GAS) genomes, while Galardini et al. (2019) used it to characterize genetic determinants of extra-intestinal virulence in *Escherichia coli*.

Even in its nascency, mGWAS have played a critical role in public health microbiology. Of particular interest is antimicrobial drug resistance which poses a significant threat to public health, especially due to the emergence of several multidrug-resistant strains worldwide (Aun et al., 2018; Wozniak et al., 2014; Frost et al., 2019). mGWAS has been crucial in identifying novel genomic markers responsible for drug resistance. In a recent study, Farhat et al. (2019) estimated heritability of resistance phenotype in 1526 *Mycobacterium tuberculosis* isolates to 11 anti-TB drugs and reported 13 non-canonical loci that were associated with resistance. Another study (Earle et al., 2016) used mGWAS to detect genes and genetic variants associated with resistance to 17 antimicrobials in 3,144 isolates from four taxonomically diverse and recombining bacterial species. The authors also confirmed a rise of over 20 times in antimicrobial resistance per drug in the *M. tuberculosis* tree, through frequent convergent evolution (Earle et al., 2016). Furthermore, mGWAS has been used to identify novel and known markers associated with HIV drug resistance (Power et al., 2016a) and genetic loci in *Plasmodium falciparum* associated with resistance to several antimalarial drugs (Wang et al., 2016). Understanding the genetic architecture of a particular drug resistance phenotype makes it possible to explore other genetically correlated (or anti-correlated) phenotypes and thus inform treatment, drug design and repositioning decisions.

**FIGURE 1:**
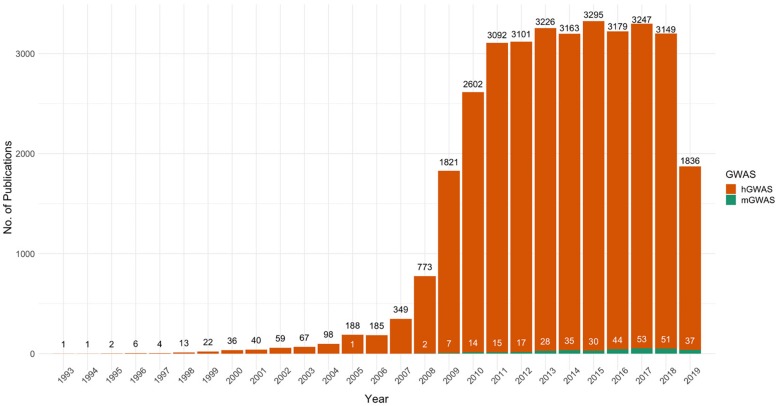
The proliferation of PubMed-indexed articles on human and microbial genome-wide association studies, 1993–2019. The figure represents results of PubMed searches for “Human Genome-wide Association Studies” and “Microbial Genome-wide Association Studies.” Trends show that the steady increase in human GWAS publications has not been mirrored in microbial GWAS. 2019 shows publications only up until end of November.

In spite of the success of mGWAS, its proliferation has remained low due to various challenges. This is evident from the almost stagnant rate of increase in mGWAS publications compared to hGWAS, which is now a fully developed research field with over 35,000 publications (Figure 1). In order to unlock the full potential of mGWAS, we need to understand the current state of the field and shed light on the bottlenecks that have stifled progress.

Microbial genomes vary widely both in terms of gene content and sequence diversity. This plasticity hampers the use of traditional single nucleotide polymorphism (SNP)-based methods for identifying all genetic associations with phenotypic variation (Lees et al., 2016). Early GWAS relied heavily on genotyping chips containing a large number of synthetic, single-stranded DNA oligonucleotides (“*oligos”*) functioning as DNA probes (Kwok and Chen, 2003; Carr, 2016). Because of the high plasticity of the genomes, the chips quickly became obsolete (Mueller, 2004). These chips also did not allow for a fine-scale correction of population structure. Genotyping chips are both expensive and restricted to mutations present in the reference genome used at its creation (Hugerth and Andersson, 2017; Read and Massey, 2014). As a result, only a few organisms like *Neisseria meningitidis* (Bille et al., 2005, 2008), *Mycobacterium tuberculosis* (Troesch et al., 1999) and *P. falciparum* (Jacob et al., 2014) with highly conserved genomes, very low rates of mutation (Dutilh et al., 2013) and that are of high global health significance were genotyped. These bottlenecks have primarily been resolved by the advent of next-generation sequencing (NGS) that offers a relatively cheap and fast solution to produce whole genomes at an unprecedented rate (Mardis, 2008; Schuster, 2008), paving the way for novel biomarker discoveries.

The need for more specialized methods has been another major bottleneck. Early studies adopted tools developed for human studies, such as PLINK (Purcell, 2007) and FaST-LMM (Lippert, 2011), to analyze microbial genomes. However, it soon became apparent that the underlying assumptions behind these tools, such as ploidy, multiple testing and population structure correction methods and tests for association (Farhat et al., 2013) were not directly applicable. For example, in a study by Sheppard et al. (2013) to identify factors responsible for adaptation of *Campylobacter* to cattle and chickens, they had to create a novel method as naively applying the Fishers exact tests would result in many spurious associations. Similarly, in a separate study by Farhat et al. (2013) to determine genes under positive selection in *M. tuberculosis (MTB)*, the haplotype-based tests could not be used as diversity in MTB mainly arises from clonal expansion and homologous recombination (Achtman, 2004) which complicate phylogenetic reconstruction. Instead, they developed PhyC, an independent test that leverages evolutionary convergence. PhyC detected 50 significant SNPs compared to PLINK’s 133 clearly highlighting the need to adapt GWAS methods to microbial genomes.

Microbial genomes further reveal a range of peculiarities that demand major feature enhancements to existing tools and new methods. For example, they are highly affected by within-host diversity (Power et al., 2016b) and phenotypic heterogeneity among others that warrant the need for new methods in order to avoid spurious results.

Several tools and methods have been developed to address these bottlenecks. To improve their usability, a number of them have been combined into automated workflows (Lees et al., 2018). Statistical and graphical overlays have also been developed to aid the interpretation of results (Jaillard et al., 2018). This enables researchers to choose the options that are suitable for their research and conform to their technical competencies and analytical platforms. As such, we anticipate an increase in mGWAS research that can then inform the discovery and development of more effective vaccines and antimicrobials for pressing infectious diseases of global health significance, such as HIV, TB, influenza, and malaria.

With the growing number of disparate tools available to perform mGWAS analyses, the choice of tool, methods or workflows presents a major challenge to biologists as there is no theoretical review of the features of existing tools or comparative analysis currently available. In this review, we discuss the prominent and promising tools and the progress that has been made in addressing the methodological challenges affecting microbial GWAS (summarized in Table 1). We also highlight the pitfalls and analytical considerations that need to be made to ensure successful microbial GWAS and the gaps that still need to be filled (Table 2) and how developers can work together to address these pitfalls and bottlenecks.

**TABLE 1:** Summary of details of prominent and promising bioinformatics tools and pipelines available for microbial GWAS.

**Software**	**Primary Usage**	**GUI**	**References**	**Implemen- tation**	**Analysis**	**Statistical Methods**	**Input**	**Output**	**User Support and documen- tation**	**Organism used for testing (Sample Size)**	**Types of phenotypes the tool can test**	**Phenotypes Tested during development**
CCTSWEEP and VENN	Commandline	No	Habib et al., 2007	Shell Scripting	∘ SNPs	Correlations	Apomorphy lists output by popular phylogenetic analysis packages PAUP, POY or TNT	Table of significant SNPs	Not available	*B. anthracis* (15)	Binary	∘ Susceptibility
GWAMAR	Commandline	No	Wozniak et al., 2014	Python	∘ SNPs, genes	∘ Mutual information ∘ Odds ratio ∘ Hypergeometric test ∘ Weighted support	A set of mutation profiles and drug resistance profiles to associate i.e., list of strains, phylogenetic tree, drug resistance profiles, list of point mutations, gene profiles and gold assocs list e.g., from TBDReamDB	Table of significant SNPs mutations with information on drug name, affected genes and methods used to determine mutation	Manual and presentation at http://bioputer.mimuw.edu.pl/gwamar/software.html	*M. tuberculosis* (173) (1398) *S. aureus*	binary	∘ Drug resistance
SEER	Commandline	No	Lees et al., 2016	C ++	∘ *k*-mers, SNPs, genes	*k*-mer counting ∘ Large studies - distributed string mining (DSM) ∘ Samples less than 5000 - fsm-lite (single core) ∘ Old datasets and not memory, DSK Fixed effects generalized linear regression including FIRTH regression	Raw fastq or assembled whole genomes	Association file with *p*-values, effect-size, direction and standard error	Extensive documentation at https://github.com/johnlees/seer/wiki/Usage	*S. pneumoniae* (3069) *S. pyogenes* (675)	Binary	∘ Drug resistance ∘ Invasive disease
Scoary	Commandline	Yes	Brynildsrud et al., 2016	Python	∘ Tests clusters of ortholog genes (COGS)	∘ Fishers exact test, binomial test and permutation test	Gene presence absence file from Roary and phenotype file	List of genes sorted by strength of association per trait	Extensive documentation on the github repo https://github.com/AdmiralenOla/Scoary	*S. epidermidis* (50) *S. pneumoniae* (200)	binary, Categorical	∘ Linezolid resistance
bugwas	Commandline, Text Editors	No	Earle et al., 2016	∘ R-package for population adjustment ∘ R, Python, C ++ end-to-end GWAS pipeline	∘ *k*-mers, SNPs, genes	Linear mixed model X2 test for *k*-mers	Raw reads in Bam or Fasta format	List of top significant k-mers annotatable by blast	https://github.com/janepipistrelle/bacterial_GWAS_tutorial/blob/master/tutorial.rmd	*M. tuberculosis* (1735) *S. aureus* (992) *E. coli* (241) and *K. pneumoniae* (176)	Binary, Categorical and continous	∘ Drug resistance
TreeWAS	Commandline, Text Editors	No	Collins and Didelot, 2018	R Package	∘ *k*-mers, SNPs, genes	∘ 3 Association tests i.e., Terminal, Simulteneous, and subsequent	∘ A phylogenetic tree infered by recombination aware approach of class phylo (optional) ∘ A genetic dataset (matrix containting binary genetic data) ∘ A phylogenetic variable (factor or vector containing binary or continous variable encoding) ∘ An ancestral state reconstruction of the genotype (matrix - optional) ∘ An ancestral state reconstruction of the phenotype (vector or factor - optional)	Set of significant loci identified data either used by or generated within treeWAS including the ancestral state reconstruction data	∘ R viginette https://github.com/caitiecollins/treeWAS/wiki	*N. meningitidis* (171)	Binary, Categorical and continous	∘ Drug resistance ∘ Invasive disease
Phenotype Seeker	Commandline	No	Aun et al., 2018	Python	∘ *k*-mers	Welch’s two-sample t-test for continous phenotype and chi- square test if binary Then a logistic or regression model is built. “Phenotype-Seeker prediction” uses the regression model generated by “PhenotypeSeeker modeling” to conduct fast phenotype predictions on input samples	Text file containing tab separated lists of; (1) sampleID’s, (2) sample FastA/FastQ file addresses and (3) sample phenotype values (one or more column).	Phenotype Seeker output gives the regression model in a binary format and three text files, which include the following: (1) the results of association tests for identifying the *k*-mers most strongly associated with the given phenotype, (2) the coefficients of *k*-mers in the regression model for identifying the *k*-mers that have the greatest effects on the outcomes ofthe machine learning model (3) a FASTA file with phenotype-specific k-mers, assembled to longer contigs when possible, to facilitate an user to perform annotation process, and (4) a summary ofthe regression analysis performed	https://github.com/bioinfo-ut/Phenotype Seeker	*P. aeruginosa* (200) *C. difficile* (459) and *K. pneumoniae* (167)	Binary and continous	∘ Drug resistance ∘ Human carriage status
Kover	Commandline	No	Drouin et al., 2016	Python and Cython	∘ *k*-mers	Set Covering Machine (SCM)	Kover matrix-generated using cover scripts	Multivariate machine learning models	∘ installation: http://aldro61.github.io/kover/doc_installation.html ∘ Tutorials: http://aldro61.github.io/kover/doc_tutorials.html ∘ http://aldro61.github.io/kover/	*C. difficile* (470), *M. tuberculosis* (110), *P. aeruginosa* (390) *and S. pneumoniae* (616)	Binary	∘ Drug resistance
PySEER	Commandline	No	Lees et al., 2018	Python	∘ *k*-mers, SNPs, genes	Fixed effects generalized linear regression including FIRTH regression	*K*-mers, SNPs and INDELs, COGs in VCF or Rtab formats	Annotated *k*-mers with gene related information in QQ, manhattan and bi-plots)	Extensive documentation and tutorial at https://pyseer.readthedocs.io/en/master/	*S. pneumoniae* (3069) *S. pyogenes* (675)	Binary	∘ Penicillin resistance
Magnamwar	Commandline, Text Editors	No	Sexton et al., 2018	R Package	∘ Gene presence-absence	Wilcoxon test, mixed and survival analysis	Core functionality requires a file that defines the orthologous gene (OG) sets and a file containing the phenotype measurements and metadata for the statistical models. Optional functions require additional datasets	Produces an R matrix containing the gene cluster identifier, *p*-values, effect size, and presence/absence pattern for each gene	Detailed example and Vignettes on CRAN and in the package, contact information available on CRAN	*D. melanogaster*	binary	∘ Triacylglyceride phenotypes
HAWK	Commandline	No	Rahman et al., 2018	C++	∘ *k*-mers	Likelihood ratio test for nested models on case-control datasets	Raw fastq whole genomes and tab seperated sample metadata file	Fasta files container significant assembled *k-*mers	https://github.com/atifrahman/HAWK	*E. coli*	Binary, Categorical	∘ Ampicilin resistance
DBGWAS	Commandline	No	Jaillard et al., 2018	Shell	∘ *k*-mers	Linear mixed model	Draft assemblies and phenotype data for a panel of bacterial strains	Phenotype associated genetic events	https://gitlab.com/leoisl/dbgwas/	*S. aureus* (9000) M. *tuberculosis* (5000) and *P. aeruginosa* (2500)	Binary, Categorical	∘ Drug resistance

**TABLE 2:** Current progress in overcoming important microbial GWAS bottlenecks and pitfalls. Most tools require additional packages or support from external software and advanced user knowledge to perform advanced analyses.

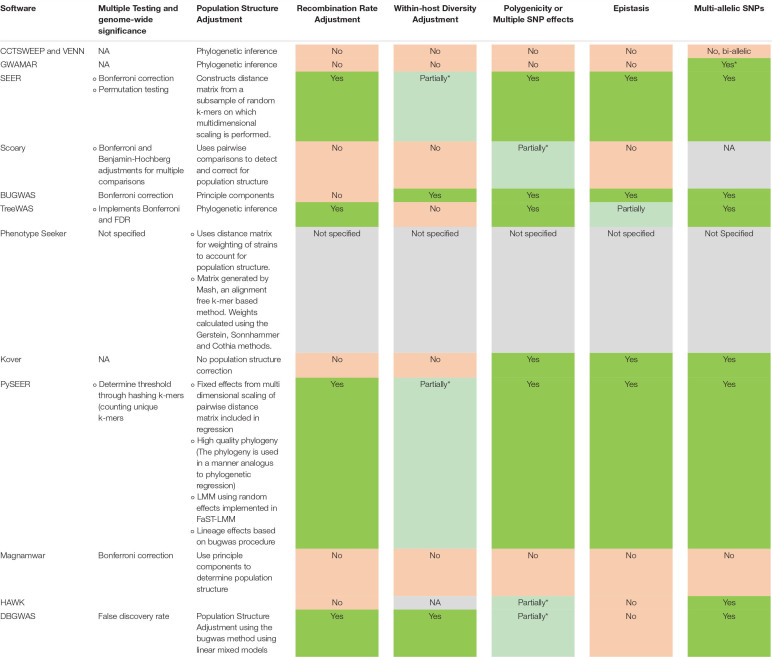

* Not supported directly but authors provide a work around so that uses can account for these changes. NA - tools functionality not affected by this feature.

## Genetic Variation in Microbes

Unlike human genetics, where the primary type of variation analysed is bi-allelic SNP variation (Wang et al., 1998), several forms of genetic variation, ranging from SNPs to the presence/absence of entire genes (Power et al., 2016b) occur in microbes (Dutilh et al., 2013; Read and Massey, 2014), implying that the expression of the phenotype of interest can be influenced by one or more of these variations. Prior knowledge of the type of variation driving the phenotype of interest in an organism is key in selecting the appropriate tool and method to use in mGWAS. However, sometimes the main type of variation responsible for a phenotype is not known *a priori*. In such instances, we recommend an agnostic approach, where all forms of variation are tested for. HAWK enables the mapping of associations to different kinds of variants using the same pipeline (Rahman et al., 2018). The three major forms of variation are:

(a) *Single Nucleotide Polymorphisms (SNPs) and INDELs*- which are point mutations or small insertions and deletions (indels) that occur within the genome of an organism (Dutilh et al., 2013) during cell duplication or transcription for viruses and bacteria. They are typically 1 to 10000 base pairs long (Weber et al., 2002; Mills et al., 2006) and can be identified by the alignment of the DNA sequence of an organism to a high-quality reference of the same strain. Microarray genotyping chips and variant calling pipelines, such as GATK (DePristo et al., 2011) and SAMtools (Li et al., 2009), are used to determine SNPs and INDELs which are then tested for association with phenotypes of interest.
(b) *Gene presence-absence* occurs when entire genes are lost or gained. Several processes are responsible for gene presence-absence. These include speciation events (Fitch, 1970) horizontal or lateral transfer of mobile genetic elements (MGEs) such as transposons and insertion sequences (IS). In bacteria, it can also be attributed to infection with bacterial phages or viruses and acquisition of plasmids or integrative and conjugative elements (ICEs) (Sobecky and Hazen, 2009; Partridge et al., 2018; Langille et al., 2010). MGEs and phages play a critical role in the interaction of the organism with its environment, for example encoding genes necessary to cope with adverse conditions or confer pathogenicity (Schmidt and Hensel, 2004). The differential expression (presence-absence) of homologous genes is a common approach applied to determine genes responsible for a given phenotype in microbial GWAS. In this approach, the core genome or genes shared by all closely related organisms, usually at the species level, are eliminated and the unique genes only present in a given species are tested for significant association to the phenotype of interest.
(c) *Copy Number Variations (CNVs) and Sequence Inversions (SIs).* They contribute to adaptation and phenotypic variation of microbes (Kirkpatrick and Barton, 2006). CNVs and SIs, like gene presence-absence, can result from acquisition of additional copies of a gene from mobile genetic elements or large-scale deletions or duplications of sections of the genome. They can also arise from speciation events.

## Phenotypes Definition

Phenotypes are the observable characteristics of microbes as a result of the interaction of their genotypes with the environment (Chibucos et al., 2014). Such characteristics include susceptibility to antimicrobials, virulence, minimum inhibitory concentrations (MICs) and host susceptibility to infection among others (Dutilh et al., 2013). Brbić et al. (2016) classified microbial phenotypes into two broad categories i.e., (a) metabolic capabilities, morphology, growth conditions and b) the ability to colonize certain ecological niches. They further collated over 424 traits associated with microbes. Phenotypes can also be classified by measurement as binary, categorical and continuous for the purpose of statistical analysis. Continuous traits may be converted into categorical values. This however, can result in ambiguous categories such as “maybe” or “mild,” which may be safely discarded or redefined as “yes or no” to increase statistical power during analysis (Dutilh et al., 2013). It should be noted that converting continuous phenotypes to categorical can be costly in terms of statistical power to detect significant associations (Altman and Royston, 2006; Power et al., 2016b) and therefore should be done with caution.

## Interpreting GWAS Results

**FIGURE 2:**
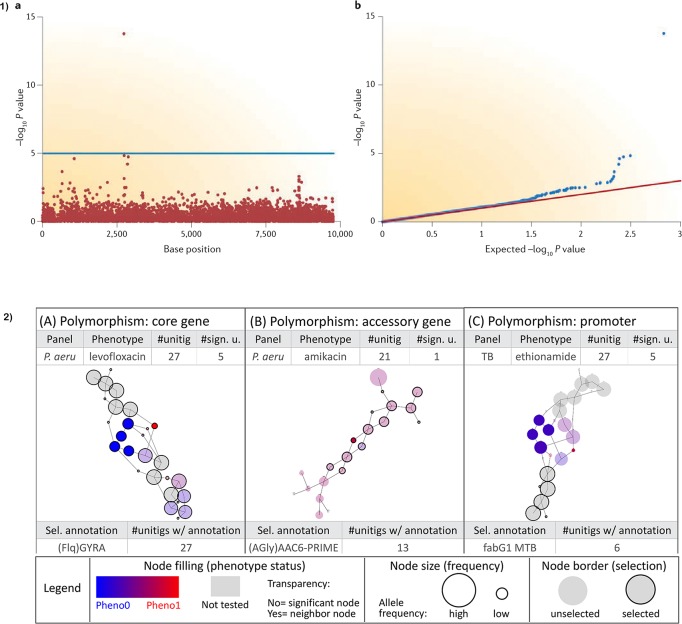
Visualization of GWAS results; (a) Manhattan and QQ plots visualizing the results. Horizontal line on the Manhattan plot shows cut-off for genome-wide significance. Only a single SNP passing genome-wide significance as shown is likely due to genotyping error as most SNPs are in linkage disequilibrium (LD) and therefore will pass the cut-off together. (b) Sample output from a DBGWAS using De Bruijn graphs to visualize unitigs. Node size relates to the allele frequency. Untested unitigs present in >99% or <1% of the strains, are shown in grey. Nodes found to be non-significant are shown with a transparency degree. Part (1) is taken from Power et al. (2016b) and (2) is adapted with permission from Jaillard et al. (2018).

The main output from conducting a traditional SNP-based mGWAS is the association file which spells out the position of the allele, allele *p*-values, the SNPs (reference and alternate), minor allele frequencies (MAF), effect size (beta for quantitative traits/odds ratio for binary traits) and standard error (SE). To help with interpretation, results are normally visualized using a Manhattan plot whose *x*-axis is the SNP position and y-axis negative log10 *p*-value or -log(*p*-value) of the SNP. A horizontal line in the plot delineates genome-wide significance threshold. All sample *p*-values above the line are considered statistically significant. A single SNP passing the significance threshold is often considered a genotyping error owing to the expectance of linkage disequilibrium (LD) (see Figure 2 Part 1b). To compare the distribution of the -log(*p*-value)s observed in the study and expected distribution under the null hypothesis, a quantile-quantile (QQ) plot is used. From the QQ plot, population stratification or polygenicity can be inferred (Power et al., 2016b). The output from methods using *k*-mers, unitigs, and gene presence-absence matrices is slightly different with additional fields representative of the specific method used. Idury and Waterman (1995) and Muggli et al. (2017) graphs provide a suitable solution for visualization of unitigs (Figure 2).

## Analytical Considerations and Pitfalls

Previous research describes in detail the important analytical considerations for microbial GWAS (Dutilh et al., 2013; Chen and Shapiro, 2015; Power et al., 2016b). Here, we provide a succinct overview to guide the researcher’s choice of tool to use and highlight the gaps that still need to be filled through current and future efforts.

Recombination rate refers to the frequency of recombination which generally varies widely among microorganisms such as bacteria and viruses and can thus limit the ability of GWAS to pinpoint specific variants responsible for the phenotype if not accounted for Didelot and Maiden (2010) and Epstein et al. (2018). In (Py)SEER, adjustments can be performed by using a recombination adjusted phylogeny (e.g., from gubbins or clonalframeML) to estimate the kinship matrix for either the fixed or Linear Mixed Model (LMM) mode, or using lineages/strains as covariates, which ignores ancestral recombination events. It is, however, important to note that determining recombination across a diverse species, which is the most common GWAS situation, is still very difficult to do accurately. DBGWAS is also able to detect and summarize recombination events at the population scale in its third step of analysis (clustering the tested features into subgraphs representing genomic regions).

There is evidence that for some pathogens within-host genetic diversity is common (Worby et al., 2014; Martin et al., 2018). It occurs as a result of evolution within the host or due to superinfections. The presence of multiple isolates from the same host and especially of clonal background can reduce power because these isolates will share large amounts of DNA due to clonal inheritance that do not confer host adaptive traits (Sheppard et al., 2013). Furthermore, it leads to non-discrete SNP calling where the frequency of an allele reflects its frequency within the host rather than the presence or absence of an allele (Power et al., 2016b). It is therefore important that mGWAS tools are able to deal with it. In existing tools such as (Py)SEER, bugwas and DBGWAS, it can be accounted for by the user providing a covariate file. Also integrating tools such as phyloscanner (Wymant et al., 2017) to infer within host diversity could improve the quality of SNP calls.

Multiple testing is a source of false positives intrinsic to GWAS. The Bonferroni correction is usually used to correct for multiple testing. However, it is overly strict for densely genotyped and imputed studies where correlations between variants exist (Power et al., 2016b) and requires much larger sample sizes in order to detect causal variants. To overcome the issue of strictness, some tools (Jaillard et al., 2018) implement the Benjamini Hochberg false discovery rate (FDR) (Benjamini and Hochberg, 1995), a less stringent method to control for multiple testing Type I errors but it has also been found to be conservative (Storey and Tibshirani, 2003) as it assumes that SNPs are independent, which is seldom true (Marees et al., 2018). Understanding the level of LD between SNPs and computing an appropriate significance threshold that is optimal for each study (Visscher et al., 2012) therefore presents a feasible and ideal solution. Existing methods for calculation of thresholds include permutation testing and spectral decomposition with the former being preferred as it is less computationally intensive (Storey and Tibshirani, 2003).

Polygenicity or multiple SNP effects on a phenotype is based on the assumption that many SNPs with small effect sizes will fail the stringent cutoff used for genome-wide significance, however, together their cumulative effect will explain a large amount of the variance in risk (Power et al., 2016b) hence providing a more powerful predictive tool than the result of a single SNP. This is particularly common with variants affecting phenotypes under moderate selection and quantitative traits, for example virulence (Fisher, 1930; Cavalli and Maccacaro, 1952; Pritchard and Di Rienzo, 2010; Laabei, 2014). Detection of unlinked, non-epistatic small effect variants affecting phenotypes is currently well handled by most tools especially tools that implement LMMs such as Bugwas and (Py)SEER. The LMM mode also gives boosted statistical power to detect associations compared to other methods. In DBGWAS, only SNPs occurring together on single unitig (haplotype) are tested together. SNPs occurring further apart will not be tested together, hence partial support. KOVERs conjunctions (logical-AND) and disjunctions (logical-ORs) models for detection of the presence or absence of *k*-mers are able to pick up the effect of multiple SNPs. Moreover, they also assign importance to each rule that quantifies how much it affects the phenotype predictions made by the model, while TreeWAS accounts for this by allowing each SNP to contribute only partially to the phenotype. Finally, HAWK reports all SNPs that present a strong effect on the phenotype without determining how much each SNP is contributing.

Microbe genomes can have multi-allelic SNPs and genes which may be responsible for the different types of phenotypes (Khachatryan et al., 2019) or code for different amino acids. The presence of multiple alleles is easily captured by *k*-mer-based methods, for example in a study analyzing *Campylobacter. jejuni*, multi-allelic *k*-mers were tested for association with host preference (Sheppard et al., 2013). In general, most of the available tools support the analysis of multi-allelic SNPs. Even tools such as HAWK and Scoary that do not perform SNP calling support the analysis. CCTSWEEP/VENN currently supports only bi-allelic SNPs.

Epistasis results when two SNPs interact or when the effect of a SNP is conditional on a broader genetic background. Disentangling epistatic effects will be key to generating viable *in vitro* models of mGWAS findings and establishing causality (Power et al., 2016b). Detection of epistatic interactions between SNPs or genes can be achieved by creating a genetic variant matrix of interaction effects which is created by multiplying the matrix of potentially interacting variants with itself. In PySEER, it can be achieved through the generality of the *–pres* input option. A program such as SpydrPick (Pensar et al., 2019) is however recommended as a better choice for detecting genome-wide epistasis for users of (Py)SEER. Also, KOVER using its conjunction and disjunction models described above is able to detect these interactions between SNPs. Bugwas and TreeWAS are also able to detect interactions between SNPs and report them to the user.

Heritability is a classical concept in quantitative genetics which represents the amount of variation in a trait which can be ascribed to genetics (and is therefore inherited between generations) versus other environmental factors (Lynch and Walsh, 1998). In mGWAS, it has been used to establish the strength of the relationship between host phenotypes and variation in microbial genomes, for example, Young et al. using heritability estimates established the presence of a strong relation between *S. aureus* genetic variation and pyomyositis, with estimated heritability at 63%. In another GWAS of human and pathogen, Lees et al. (2019) show that human variation explains almost half of variation in susceptibility to pneumococcal meningitis and one-third of variation in severity while Pneumococcal genetic variation explains a large amount of invasive potential (70%), but had no effect on severity. Determining heritability before performing GWAS is important to ensure that a substantial amount of variation is actually as a result of genetic variation. Heritability estimation is currently done as an independent step and not as part of the pipelines reviewed in this article.

In most phylogenetic analyses, beyond heritability, accounting for the heterogeneity in evolutionary patterns across sites is particularly important (Wang et al., 2019). Partitioning remains the most commonly used method for accounting for variation in the rates and patterns of molecular evolution among sites in phylogenetic analyses. An inherent obstacle in partitioned phylogenetic analyses is the choice of an appropriate partitioning scheme (Frandsen et al., 2015). Efficient partitioning schemes for small datasets are described by Lartillot et al. (2009) and Wu et al. (2013) and for large datasets by Frandsen et al. (2015). Genomic partitioning can help determine whether the SNPs in different genomic regions or lineages play different roles in trait heritability and which region is more responsible for phenotypic variation (Lees et al., 2017; Wei et al., 2019). Heritability and genomic partitioning are therefore key components of a phylogeny-based microbial GWAS workflow.

Sample size, unlike in human GWAS, mostly poses a subtle problem as most variants in microbial genomes are under strong selection and therefore present large effect sizes even with small datasets. Subtle traits resulting from many low effect variants, however, require larger sample sizes in order to detect significant associations (Power et al., 2016b). For phylogeny-based workflows, allele counting methods require larger sample sizes compared to homoplasy counting methods (Chen and Shapiro, 2015). In such circumstances, tools such as Scoary (Brynildsrud et al., 2016), that are inherently lightweight may not apply. It is also important to note that, as with any GWAS study, power loss is greatest when the number of variants is high and the number of samples is small. Prior calculation of the number of samples required to reach sufficient power is thus an important first step to determine that the selected tool is suitable.

## Microbial–Based GWAS Tools

Traditional microbial-based GWAS tools can be broadly categorized into three categories: (a) phylogeny, (b) non-phylogeny and (c) hybrid tools that implement a combination of statistical and phylogenetic methods. An emerging fourth group that is gaining traction comprises tools that apply machine learning to the prediction of phenotypes from genotype data. The main limitation of machine learning tools is their performance however, as more whole genome sequences become available, their predictive accuracy is expected to improve. In this review we profile eleven traditional microbial GWAS tools and two machine learning applications.

(1) *CCTSWEEP and VENN* (Habib et al., 2007) use phylogenetic trees to find correlations between SNPs that are statistically significant. VENN operates on apomorphy lists produced by popular phylogenetic tools. A major limitation of VENN is that it only works well for a large number of SNPs, where the number of branches over which change is occurring is modest. In contrast, CCTSWEEP works even when there are no SNPs that are completely penetrant with the phenotype of interest. CCTSWEEP implements a modified version of the Maddison’s concentrated changes test (CCT) (Maddison, 1990; Habib et al., 2007).

An advantage of both VENN and CCTSWEEP is that they consider missing data using character optimization which other methods simply ignore. The tools have been used to find SNPs correlated with resistance to *Bacillus anthracis* in inbred mouse strains. VENN successfully identified 11 SNPs from 4 chromosomes (Habib et al., 2007) while CCTSWEEP identified 12 SNPs in chromosome 11 (Habib et al., 2007). The major limitation with CCTSWEEP is that it calculates the correlation between binary variables only.

(2) *GWAMAR is a* tool specifically developed to detect drug resistance-associated mutations in bacteria (Wozniak et al., 2014). It computes several statistical scores, including mutual information, odds ratio, hypergeometric test, weighted support and the tree-generalized hypergeometric score (TGH), which is a modification of the CCTSWEEP score method. Developers of GWAMAR have tested the tool on two *M. tuberculosis* datasets. The results of both case studies demonstrated that the tree-aware methods (weighted support and TGH) performed better than those that did not include phylogenetic information. Using GWAMAR on the two datasets also allowed for the identification of novel mutations putatively associated with drug-resistance (Wozniak et al., 2014). However, despite the promising results, the tool has the following limitations: (i) it does not take into account or predict epistatic interactions between mutations, and (ii) it only takes genomic changes into account and ignores levels of gene expression.

(3) *Sequence Element Enrichment (SEER) Analysis* is a *k*-mer based tool that counts variable length *k*-mers using a distributed string-mining algorithm implemented in C ++ (Lees et al., 2016). It provides options to correct for clonal population structure and performs well on large datasets spanning tens of thousands of genomes, both assembled and unassembled. It has been developed as a stand-alone pipeline that takes as input either *de novo* assembled contigs or raw read data. SEER has been tested on *S. pneumoniae* and *Streptococcus pyogenes* datasets and was able to successfully identify previously characterized resistance determinants for several antibiotics in the former and unearthed novel factors related to invasiveness in the latter. A major distinction from other tools is that it was built with meta-analyses in mind i.e., the output includes effect sizes, direction, and standard error. These can be used directly with existing software to meta-analyze all overlapping *k*-mers. A major challenge with SEER is its complexity. It requires the user to execute several steps and install many system-level dependencies for compilation and installation (Aun et al., 2018).

(4) *Scoary* is a tool that scores the components of the pan-genome (i.e., the full complement of genes in a clade) for associations to observed phenotypic traits while accounting for population stratification (Brynildsrud et al., 2016). It does so with minimal assumptions about the evolutionary process. A major advantage of Scoary is that users do not need to experiment with ill-informed mutation rate parameters or inform the program about population structure as this information is directly inferred from input data. Scoary validates results using a *post hoc* label switching permutation test. It is intended to be an intuitive, fast and platform-independent tool. This is achieved by providing a graphical user interface and easily understandable results. A major limitation of the tool is that it is not designed to handle large sample sizes spanning thousands of bacterial genomes. Scoary supports binary or categorical phenotype data. Quantitative phenotypes require binning into distinct categories. It implements pairwise comparisons to control for spurious associations. These comparisons account for fine-scale genetic differences and phylogenetic clustering. However, they are also notorious for discarding large volumes of valuable data (Collins and Didelot, 2018). Scoary was able to successfully predict *cfr*, a well-known gene associated with high-level resistance to the antibiotic linezolid, and two other plasmid genes (*pin*E, *cue*R) at genome-wide significance with a modest sample size of 21 isolates (Brynildsrud et al., 2016).

(5) *Bugwas* is a robust bacterial GWAS end-to-end pipeline implemented in R, Python, and C ++ (Earle et al., 2016). It is capable of performing SNP, *k*-mer and gene differential analysis. Bugwas uses principal components and linear mixed models (LMM) to identify and correct for population structure. The LMM’s are implemented using Genome-wide Efficient Mixed Model Association (GEMMA) (Zhou and Stephens, 2012), a fast software toolkit for the application of LMM’s to GWAS. An independent R package that implements the Bugwas method to control for population structure is also available on GitHub^1^. Bugwas is also able to detect polySNP and polygenic effects when multiple low effect variants are responsible for the phenotype and not a single high effect variant. It takes into consideration both locus effects and lineage effects without losing power to detect significant variants. Bugwas was used to determine resistance to 17 antimicrobials in 3,144 isolates across the major pathogens *M. tuberculosis, S. aureus, E. coli*, and *Klebsiella pneumoniae* (Earle et al., 2016). It successfully identified genuine causal loci or regions in physical linkage with those loci for antimicrobial resistance in 25/26 cases for the SNP, gene presence-absence and *k*-mer approaches after controlling for population structure. Additionally, Suzuki et al. (2016) used Bugwas to identify a horizontally transferred surface adhesin gene in *Acinetobacter baumannii* and a specific section of the gene that appeared to accumulate variations across the different branches of the carbapenem-resistant strains.

(6) *TreeWAS* is a phylogenetic method implemented in an R package that measures the statistical associations between a phenotype and genotype at all loci while correcting for the confounding effects of clonal population structure and homologous recombination without losing statistical power to detect associations (Collins and Didelot, 2018). The treeWAS package supports binary phenotype data, discrete interval (categorical) and continuous phenotypic data. It is applicable to both bacterial and viral genetic data from both core and accessory genome. Additionally, it supports integration with ClonalFrameML (Didelot and Wilson, 2015), a software package that performs efficient inference of recombination in bacterial genomes. The package has been tested on *Neisseria meningitidis* to identify penicillin resistance and invasive disease-associated variants (Collins and Didelot, 2018). For penicillin resistance, measured both as a binary (resistant vs. susceptible based on minimum inhibitory concentration (MIC) threshold) and continuous (ranks of MIC values) variable, no genes were found to be associated. Instead, several significantly associated SNPs were identified in the NEIS1753(*penA*) gene that encodes penicillin-binding proteins and in three additional genes. For invasive meningococcal disease, it located 12 genes and 7 SNPs that were significantly associated. Considering the complexity of the invasiveness phenotype, the results show that the package is well suited for detecting loci with subtle and complex phenotypes which may not be entirely determined by genetic factors. A limitation of TreeWAS is that being implemented in R requires users to have basic knowledge of the programming language, which often may not be the case for many researchers.

(7) *PhenotypeSeeker* is a machine learning tool for the prediction of host-phenotypes from associated bacterial genotype data (Aun et al., 2018). The software identifies phenotype-specific k-mers, generates statistical models based on them and uses the models to predict host phenotypes from bacterial isolates. The models generated can also be used in other machine learning applications to predict the associated phenotype. PhenotypeSeeker is made up of two complementary modules – one for modeling (*PhenotypeSeeker modeling*) and another for prediction (*PhenotypeSeeker prediction*). The modeling module applies the Welch’s two-sample *t*-test if the phenotype is continuous and a chi-squared test if it is binary. It then constructs a regression or linear model which is consumed by the prediction model and used to predict phenotypes. It optionally performs weighting of strains using a distance matrix of the strains to account for population structure. The final output is a complete list of statistically significant candidate variations. It is both easy to install and easy to use, requiring just two commands to run a complete analysis. Only searching for the *k*-mers in the regression model makes PhenotypeSeeker very fast. However, in the presence of novel mutations when using the models, this would become a limitation.

(8) *Kover* is a reference-free method for the identification of biomarkers that relies on the *k*-mer representation of genomes and the set covering machine learning algorithm to produce intelligent multivariate models (Drouin et al., 2015, 2016). The models can be consumed by other tools or visualized and explored further to determine the underlying causal biomarker using existing tools, such as nucleotide blast and Unipro UGENE. It is capable of identifying SNPs, indels and large-scale genomic rearrangements. Like PhenotypeSeeker, Kover uses a machine learning approach that seeks a computational model of a sparse and accurate matrix of the fewest *k*-mers required to predict a phenotype of interest. Such a method helps reduce the computational overhead by eliminating less informative *k*-mers. Kover is implemented in Python. The need to manually install some dependencies can make it challenging for non-technical users. However, the tool is well documented and does not require a machine learning background. Several models that are readily applicable are available on the Kover website. A major limitation of the Kover models is their inability to predict categorical variables with more than two levels, for example, adding intermediate antimicrobial resistance as a third category (Rodloff et al., 2008; Jeukens et al., 2017). Additional work on the Kover implementation needs to be done to improve the sensitivity of the algorithm through the inclusion of prior knowledge of population structure (Drouin et al., 2016).

(9) PySEER is a direct Python reimplementation of SEER (see above) with several enhancements (Lees et al., 2018). It uses generalized linear models to test for associations between each *k*-mer (i.e., short DNA string of length *k*, where *k* is small number typically between 3 and 100 base pairs) and phenotype. To control for population structure, it performs multi-dimensional scaling of a pairwise distance matrix and the components are included as fixed effects in the model. After adjusting for multiple testing, significant *k*-mers can be mapped to a reference annotation to find regions of the genome associated with the phenotype. PySEER also allows for testing of association of SNPs and indels called against a reference genome and implements machine learning prediction with a regularized regression approach/elastic net. Interactive visualizations are generated using an implementation of Phandango (Hadfield et al., 2017). Finally, the application can estimate possible lineage effects based on the procedure used in bugwas (Earle et al., 2016). Unlike its predecessor SEER, PySEER can be installed via conda which is fast and eliminates the need to install dependencies manually. However, as a limitation the user needs to have a good understanding of the command-line to successfully execute all the commands and prepare the relevant inputs.

(10) *MAGNAMWAR* is an R package for assessing genotype-phenotype relationships using orthologous genes in bacteria (Sexton et al., 2018). The package can be used to define the genetic relationship between bacterial genomes or metagenomes and any organismal phenotype, for example, it has been used to identify bacterial genes associated with variation in *Drosophila melanogaster* (fruitfly) phenotypes (White et al., 2018) which though outwardly different from humans, shares over two-thirds of its genes with humans (Greenspan and Dierick, 2004). This, coupled with their rapid reproduction makes them an ideal substitute for humans in research labs (Pandey and Nichols, 2011). It consumes as input orthologs produced by OrthoMCL (Fischer et al., 2011) and a phenotype file containing phenotype measurements and metadata for the statistical models. It implements multiple robust statistical analyses, including mixed and survival models as well as the Wilcoxon test for association. The software also provides the functionality to perform functional annotation of genes. Genes that are not functionally classified are clustered into phylogenetic distribution groups (PDGs). PDGs are a useful way to analyze genes that lack functional annotation. Homologous genes from the closely related strains are grouped together and association testing performed on these genes. MAGNAMWAR simplifies the pre-formatting and analysis steps, and the graphical presentation of the data. Magnamwar is limited to gene-presence absence and therefore cannot be used to analyze associations with other forms of variation.

(11) *Hitting Associations with k-mers (HAWK) is a k*-mer based tool that uses logistic regression to determine *k*-mers which are significantly associated with a phenotype of interest (Rahman et al., 2018). It has been developed in C ++ and has implemented multi-threading in order to speed up the analysis.

The tool has been tested on an *E. coli* dataset for ampicillin resistance. It uses principal component analysis to detect and correct population structure. HAWK accepts raw FASTQ files as input and requires the reads for each sample to be in a separate directory. Using the same pipeline, one is able to map associations to different types of variants including SNPs, INDELs and structural variations such as copy number variations (CNVs). Future work of interest to the developers that is likely to add value to the community of users include modeling stochasticity in counts, incorporating confounders as well as extending the approach to quantitative phenotypes as future work.

(12) *De Bruijn Graph GWAS (DBGWAS)* is a freely available *k*-mer based tool that produces interpretable genetic variants associated with distinct phenotypes (Jaillard et al., 2018). The main goal of DBGWAS is to bridge the gap between SNP and *k*-mer-based GWAS. The former is unable to cover complete genomic variation and the latter produces complex and hard to interpret results while doing so. In order to bridge the gap, it uses De Bruijn (DBGs) graphs (Idury and Waterman, 1995; Muggli et al., 2017) i.e., a set of vertices representing the *k*-mers connected by edges to compact and abstract the complexity behind *k*-mers while providing a relatively easy to understand representation of the results. Compacted DBGs (cDBGs) eliminate local redundancy, reflect genome variations, and characterize the genomic environment of a *k*-mer at population level. It takes as input a set of contigs and phenotype data. It relies on bugwas, to test significant associations between unitigs and phenotypes.

An added advantage of the DBGs is their ability to accommodate more complex disparities, including rearrangements, insertions, and deletions. DBGWAS provides a web-based interface where users can further explore the results using interactive visualization. The key features of DBGWAS, reported by Jaillard et al. (2018)., are that (i) it identifies and graphs both local polymorphisms and mobile genetic elements (MGE), (ii) it reports expected variants without prior knowledge, (iii) it extracts novel variants, (iv) it provides an interpretation of *k*-mer based GWAS and (v) it is memory efficient and can scale to very large datasets. To perform GWAS, DBGWAS uses the bugwas (Earle et al., 2016) method. DBGWAS also uses GEMMA (Zhou and Stephens, 2012) to generate a relatedness matrix that is used to correct for population structure. Compiling and installing DBGWAS can be challenging to non-technical users. Furthermore, the use of *De Bruijn* graphs are relatively new and therefore interpretation of output can be challenging.

## A Note on Viruses, Fungi and Protozoans

All tools discussed so far have only been tested on bacteria, however, many of the challenges they address also affect viruses, fungi and protozoa, which implies that these tools should be applicable to them as well. Several arguments can, however, be advanced for the paucity of tools, methods and studies focusing directly on these organisms. These include; (a) the need for enough sequences for a well powered GWAS. (b) high variability of viral genomes, especially RNA viruses (Duffy, 2018; Renner and Szpara, 2018) causing major deviation from reference genomes; and (c) the continuous emergence of viral genomes (Rose et al., 2016). In spite of these challenges, GWAS has been successfully applied to viruses. For example, Bartha et al. (2013) in a genome-to-genome study of human and HIV viral genomes tested for association between host DNA polymorphisms, HIV-1 sequence variation and plasma viral load and observered significant SNP association to 48 HIV-1 amino acid variants. In another study, Ansari et al. (2017) performed a genome-to-genome interaction analysis of 542 individuals with hepatitis C virus (HCV) to identify alleles in human genes driving viral polymorphisms and found that IFNL4 genotypes determine HCV viral load. Finally, Power et al. (2016a), using GWAS were able to identify five polymorphisms that led to amino acid changes in HIV and highlighted the potential of GWAS to identify epistatic interactions.

Protozoa and fungi on the other hand, have highly conserved genomes and very low mutation rates (Long et al., 2018) making it possible to apply the tools developed for human studies with better results than viruses and bacteria. Past fungal and protozoa studies have therefore mostly relied on the software tools developed for human studies such as PLINK (Purcell, 2007), Tassel (Bradbury et al., 2007) and GAPIT (Lipka et al., 2012) that support analysis of haploid genomes and complex traits or on custom scripts created by the study teams (Lipka et al., 2012).

## Guidelines for Tools Selection and Parameter Optimization

With a large number of existing tools, and several others that are still in development, it can be quite challenging for users to determine which one is most suitable for their research. It can also be a non-trivial problem to determine optimal parameters to use in order to guarantee the best results. Below we present the important features and parameters that researchers must consider.

(1) Select a tool that supports the analysis of all/most forms of variation as they offer the advantage of testing multiple hypotheses. This is important because a single isolate can be affected by multiple forms of variation. For example, it can acquire SNPs, indels as well as MGEs. For such an isolate, testing only for SNPs misses out MGEs. And yet these MGEs could potentially be the driving force behind the phenotype (Dutilh et al., 2013). *k*-mer based tools are able to detect all forms of variation making them prime candidates. The main challenge when working with *k*-mers however, is that they are less compact than SNPs and thus require additional computational resources to process (Drouin et al., 2015). In the case of machine learning, the large number of genomic features compared to genomes also implies a higher likelihood of overfitting i.e., learning random noise patterns that can lead to poor generalization performance (Drouin et al., 2015). Majority of the *k*-mers are usually uninformative, occur simultaneously and are highly correlated. *k*-mers have thus been superseded by uniquely assemblable contigs (unitigs) which comprise overlapping fragments that together spell a common sequence and do not overlap fragments with sequences that dispute, or contest, the common sequence. Each unitig contains on average about 30 fragments. There are 100 times fewer overlaps between unitigs than overlaps between fragments (Myers et al., 2000). Unitigs remove redundancy from *k*-mers by collapsing all nodes representing the same sequence into a single node and branching nodes to show sequence variation. The results from *k*-mer/unitigs analysis also tend to be challenging to interpret. However, Jaillard et al. (2018) have devised a clever use of *De Bruijn* graphs to aid the effective visualization and interpretation of results. *S*everal tools including PySEER, DBGWAS, HAWK and others now support the compaction of *k*-mers into unitigs.

(2) While it is important to control for population structure in microbial GWAS, power to detect significant associations is lost using some methods. Prominent methods used to control for population structure include clustering, linear mixed models (LMM) and, for more clonal species, phylogenetic relatedness. The effect of recombination on several microbial organisms makes phylogenetic methods less effective and reduces their power to detect associations. In selecting a pipeline, we recommend one that implements LMMs as they offer biological insights at both locus and lineage specific levels. It will also identify groups of loci which are collectively significant, even though individually insignificant, without sacrificing the power to detect locus-specific associations. When the sample used is homogenous, the effect of population structure is less pronounced (Power et al., 2016a). Performing association testing with and without population structure correction and assessing the difference (Earle et al., 2016) would help recover power that may be lost during population structure adjustment in some methods. When using LMM based methods, Jaillard et al. (2018) noted that logistic regression based tests have less power, compared to the Poisson distribution test.

(3) Inconsistent ordering of samples between variant call, phenotype file, and population structure adjustment can result in spurious results, especially among tools that implement LMMs. Unfortunately, several tools do not check ordering and therefore leave much room for error. We recommend using a tool that automatically checks for inconsistent labels and notifies the user if they occur. Of the tools that we reviewed, PySEER was able to automatically match labels and report the intersection of samples used (Lees et al., 2018).

(4) The length of *k*-mer used impacts speed and accuracy inversely. Longer lengths increase the sensitivity of the test and guarantee more accurate results. However, they are also associated with a significant increase in the amount of memory and processor usage (Aun et al., 2018). Most studies (Drouin et al., 2015; Earle et al., 2016), especially in bacteria, have used and recommend a length of 30 to 100 bp. Aun et al. (2018) performed accuracy tests and the results suggested that a length of 13 bp should be sufficient. Drouin et al. (2015) performed further experiments with *k*-mers of lengths 11 to 99 bps and also found no significant variation in accuracy, affirming their findings. Pilot experiments by Jaillard et al. (2018) reveal that a *k*-mer length of 31 produced the best results when retrieving known markers. The results indicate that an optimal k-mer length of 31 which can be lowered down to 11 to minimize computational resources or raised up to 100 to maximize accuracy. Users need to experiment with a range of values and carefully select a length most suitable for the genome of interest. The length of k-mer used is therefore often left as a user-defined parameter in tools implementing the *k*-mer approach. Tools with heuristics to automatically determine the most optimal length for the user will ensure the best results in a shorter time frame. Other important factors that need to be considered when deciding on the value of *k* include assembly quality, complexity of the input genomes, or presence of repeats.

(5) Converting continuous or quantitative phenotypes into categorical values (binning) can be costly in terms of power to detect significant associations (Power et al., 2016b), We recommend that tools that support the analysis of quantitative phenotypes be used when the phenotype under investigation is quantitative. However, binning remains an option in the event that the most suitable tool chosen only supports binary and categorical variables (Read and Massey, 2014). On the contrary, tools that perform well on quantitative phenotypes might not necessarily do so for binary phenotypes due to the inherent assumption of constant residual variation especially in tools based on linear mixed models. Users therefore need to carefully choose the tools based on the phenotype that is supported.

## Proposed Microbial GWAS Workflow

**FIGURE 3:**
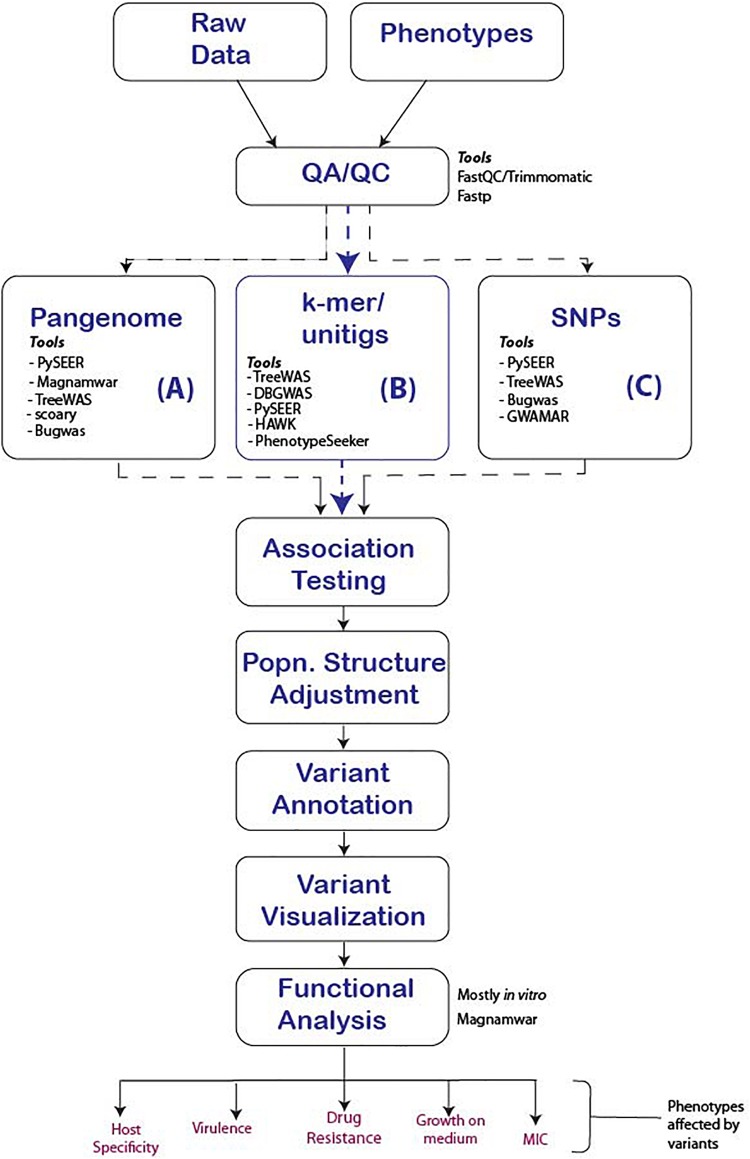
Proposed GWAS workflow reflecting major steps that should be followed for the successful conduct of microbial GWAS. We present three options to consider for any given study and tools that support the option. (A) Uses the pan-genome approach to determine unique genes in related Genus, species or strains that are significantly associated which the phenotype, (B) The use of *k*-mers, suitable in events where indels and genes are responsible for the phenotypes of interest and finally (C) SNP association, which is effective for strain-level analysis were point mutations are responsible for the phenotype of interest.

The significant and systemic genomic differences between human and microbial genomes call for substantial adaptations of older, human GWAS workflows to microbial GWAS. Here we propose a general workflow and highlight the major steps critical to a study’s success (Figure 3).

## Recommendations and Future Directions

Even though copy number variants and structural inversions have been shown to be quite frequent in some microbes and to contribute significantly to phenotypic variation, methods to perform GWAS on them remain underexplored as compared to gene presence-absence or SNPs and INDELS (Brynildsrud et al., 2015). A well-established method for association testing such as logistic regression is unable to detect association if cases have two copies of an allele against one copy in controls (Jaillard et al., 2018). Developing methods for association testing of gene copy number with phenotypes is clearly a high-priority research area.

Machine learning enables the prediction of phenotypes from genomic data as new data is made available. In this article, we cite three tools (PySEER, Kover and PhenotypeSeeker) currently implementing machine learning methods. Models created by Kover were found to have error rates as low as 10% (Drouin et al., 2016). Some of the advanced machine learning techniques currently implemented in the field of microbial GWAS include Set Covering Machines (SCM) (Marchand and Shawe-Taylor, 2002), Classification and Regression Tree (CART) decision trees (Marchand and Shawe-Taylor, 2002) and Linear Support Vector Machines (LSVM) (Burges, 1998). We anticipate an influx of tools supporting machine learning as openly accessible training data becomes increasingly available. An important consideration in machine learning and prediction for microbial GWAS is data set design and the influence that clonally related samples sharing a phenotype can have on the patterns identified by machine learning models. For example, including related isolates that are epidemiologically linked can result in significantly different results (Wheeler, 2019).

Long range linkage disequilibrium (LD) is a common phenomenon in microbial genomes (Mueller, 2004). It occurs when short sequence blocks of DNA are replaced during homologous recombination, removing variants in short LD and leaving variants further apart in LD. The presence of long-range LD is a major confounding effect in microbial GWAS that makes the identification of causal variants problematic. Testing without accounting for LD can also result in overweighting of redundant information thus inflating the effect size of a given variant (Yang et al., 2014). LD is usually evaluated by comparing the distribution of the pairwise distance between the allelic profiles. Corrections for LD can be done through a kinship matrix representing the average amount of LD between samples. While LD might make discovery power higher by linking other variants into a test, in microbial genomes, it usually severely limits mappability of associations due to its range. We recommend more work to be done toward correcting LD both in existing and new tools.

Pre-processing of raw data prior to performing any form of analysis is an important step to avoid spurious results. Many of the tools presented here depend on pre-processed results from other tools implying, that burden of ensuring appropriate input is left on the user. Extending these tools to integrate pre-processing tools for raw sequencing data through transparent calls to existing software or improved implementation of existing methods will greatly enhance the utility of the tools. In a similar manner, integration or implementation of post GWAS methods will also contribute immensely to the utility of existing tools. For example, MAGNAMWAR (Sexton et al., 2018), provides the functionality to perform functional annotation of its results.

Finally, we recommend the development of methods to improve the power and precision with which polygenic effects are detected and measured as an important future direction. The presence of these effects can be determined by the genomic inflation test and inferred from the Q-Q plot showing the difference between the expected and observed *p*-values (Power et al., 2016b) or using LMMs (Yang et al., 2014).

## Collaboration and Community Engagement

Successful development of tools and methods is a direct result of collaborative development between the software development community and their user communities. GitHub^2^, GitLab^3^ and other open source code repositories have emerged as powerful tools for collaboration. For example, nine of the tools reviewed have their code repositories on GitHub or GitLab and these also stand out as the most prominent solutions currently available. The success of these tools can be attributed among others to the useful interaction between users and developers through issues filed.

GitHub issues is one of the important features available to facilitate interaction between users and developers concerning the tools. Through this feature, users can request feature enhancements or new features, clarification on existing functionality and report bugs that they come across while running the tools. Users are encouraged to file more issues that can help improve the solutions available.

For the developers and users with technical skills, the pull request feature on GitHub provides the functionality to modify and share their contributions to a project. Pull requests remain fairly rare in bioinformatics projects. With most repositories having under four direct contributors, this feature presents a great opportunity to increase the utility of tools. As of this writing, only one tool (HAWK) had a single pull request where a user shares an improvement to the countKmers script. To improve the utility of existing tools, we recommend more collaborative efforts among the developer community.

Furthermore, we encourage open data sharing to improve the quality of testing and thus solve the problem of overfitting of tools to specific datasets or organisms. For machine learning, data sharing enables training of models that predict anti-microbial resistance (AMR) phenotypes without relying on a database of preexisting AMR genes or mutations (Nguyen et al., 2018). We recommend depositing of raw sequences in the sequence read archive (SRA), an international public archival resource for next generation sequencing data (Leinonen et al., 2011) and publishing the accession numbers. Phenotypic data, data simulation scripts and analytical results can be shared on zenodo^4^ and GitHub which are free and reliable general-purpose, open-access platforms designed for scholars and researchers.

## Conclusion

Significant strides have been made to advance the field of microbial GWAS. Several tools and methods have been developed targeting the analysis of microbial genomes however, the need for a complete, freely available and easy to use tool for microbial GWAS still remains. Biological researchers and software developers will need to work together to achieve this important cause.

## Author Contributions

JS and TO conceived and structured the manuscript. All authors generated the content and wrote the manuscript.

## Conflict of Interest

The authors declare that the research was conducted in the absence of any commercial or financial relationships that could be construed as a potential conflict of interest.

## Footnotes

1 https://github.com/sgearle/bugwas

2 https://github.com

3 https://gitlab.com

4 https://zenodo.org/
